# Lymphangiome kystique du mésocolon: à propos d’un cas

**DOI:** 10.11604/pamj.2024.48.144.35940

**Published:** 2024-08-02

**Authors:** Abdelkrim Chetibi, Kamel Allal, Mustapha Saidani

**Affiliations:** 1 Service de Chirurgie Générale et Oncologique, Centre Hospitalier Universitaire de Beni-Messous, Alger, Algérie

**Keywords:** Lymphangiome kystique, mésocolon, exérèse, anatomopathologie, cas clinique, Cystic lymphangioma, mesocolon, excision, disease, case report

## Abstract

Le lymphangiome kystique (LK) intrapéritonéal est une pathologie bénigne malformative rare, d'origine lymphatique, sa symptomatologie clinique est très diversifiée. Le diagnostic est évoqué par l'imagerie et confirmée par l'histologie. Nous rapportons ici le cas d'une femme âgée de 54 ans, consultant pour des douleurs abdominales chroniques sans retentissement sur l'état général. Le bilan radiologique est revenu en faveur d'une formation kystique mésentérique bénigne pouvant être compatible avec un lymphangiome kystique. Une exérèse chirurgicale complète de la masse kystique après ponction-aspiration est pratiquée par laparotomie médiane, les suites postopératoires sont simples. L'examen histologique de la tumorectomie permet de confirmer le diagnostic d'un LK du mésocolon. À 12 mois, la patiente est asymptomatique et aucune récidive n'est observée.

## Abstract (en)

Intraperitoneal Cystic Lymphangioma (CL) is a rare benign malformative disease of lymphatic origin, with a wide range of clinical symptoms. Diagnosis is based on imaging tests and confirmed by histology. We here report the case of a 54-year-old woman presenting with chronic abdominal pain, with no effect on general condition. Radiological assessment showed a benign mesenteric cystic formation consistent with a cystic lymphangioma. Complete surgical excision of the cystic mass after puncture-aspiration was performed via midline laparotomy, with an uncomplicated postoperative course. Histological examination of the lumpectomy confirmed the diagnosis of LK of the mesocolon. At 12 months, the patient was asymptomatic and no recurrence was observed.

## Introduction

Le lymphangiome kystique (LK) du mésentère est une tumeur congénitale malformative du système lymphatique, se révélant le plus souvent en bas âge et atteignant dans la majorité des cas la région cranio-faciale, cervicale ou thoracique [1]. Néanmoins, des localisations intra-abdominales telles que le mésentère et le rétropéritoine sont possible, mais tous les organes peuvent être touchés. La symptomatologie la plus révélatrice sont les douleurs abdominales et/ou une masse abdominale. Le diagnostic est évoqué par l'échographie et le scanner, et est confirmé par l'histologie [2]. Le traitement de référence est la chirurgie. Le suivi est basé sur l'examen clinique et l'échographie abdominale. Il est donc nécessaire de faire le diagnostic de cette masse afin d'éviter la survenue d'une complication abdominale (infarctus mésentérique, volvulus, ischémie intestinale, etc.)

## Patient et observation

**Information sur la patiente:** c'est une patiente âgée de 54 ans, aux antécédents de sarcoïdose en 2004 traitée et guérie, qui consulte pour des douleurs abdominales diffuses sous forme de sensation de gène évoluant par épisodes sans troubles du transit, ni fièvre, ni syndrome inflammatoire biologique avec un état général bon.

**Examen clinique:** il est normal chez notre patiente, hormis une masse abdominale palpable.

**Figure 1:**
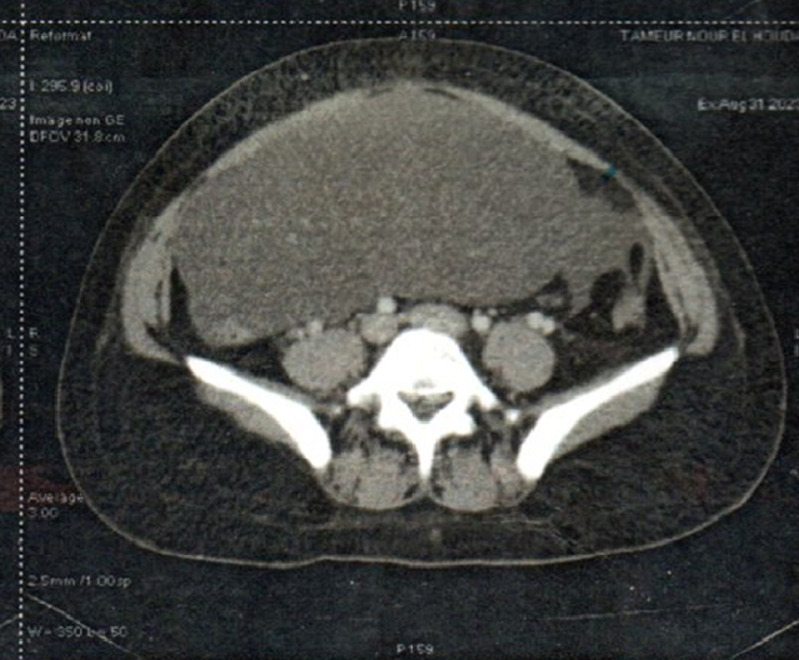
coupe axiale d'un volumineux lymphangiome kystique du mésocolon

**Démarche diagnostique:** une échographie abdomino-pelvienne est réalisée révélant une formation kystique à paroi fine, médiane, présentant quelques cloisons à contenu anéchogène, mesurant (148 x 141 x 60 mm). Le bilan d'extension complété par une tomodensitométrie (TDM) abdomino-pelvienne est revenu en faveur d'une formation liquidienne mésentérique à paroi fine, hypodense, à cloisons fines ne prenant pas le produit de contraste, limitée en dedans par l'estomac, en avant et en arrière par la grêle et le colon (Figure 1). Au terme de ce bilan clinique et radiologique, le diagnostic d'une formation kystique bénigne pouvant être compatible avec un lymphangiome kystique a été posé. Une imagerie par résonance magnétique (IRM) abdomino-pelvienne confirme la formation multikystique, mesurant (183 x 96 x ×200 mm) sans portion solide, ni végétations, développée au dépend du mésocolon. La biologie sanguine ainsi que les marqueurs tumoraux sont normaux.

**Figure 2:**
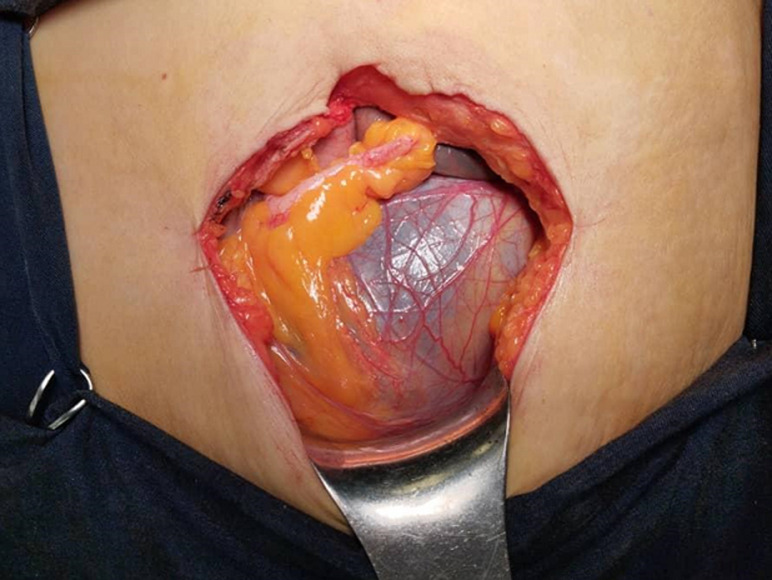
aspect per-opératoire d'un masse kystique du mésocolon

**Figure 3:**
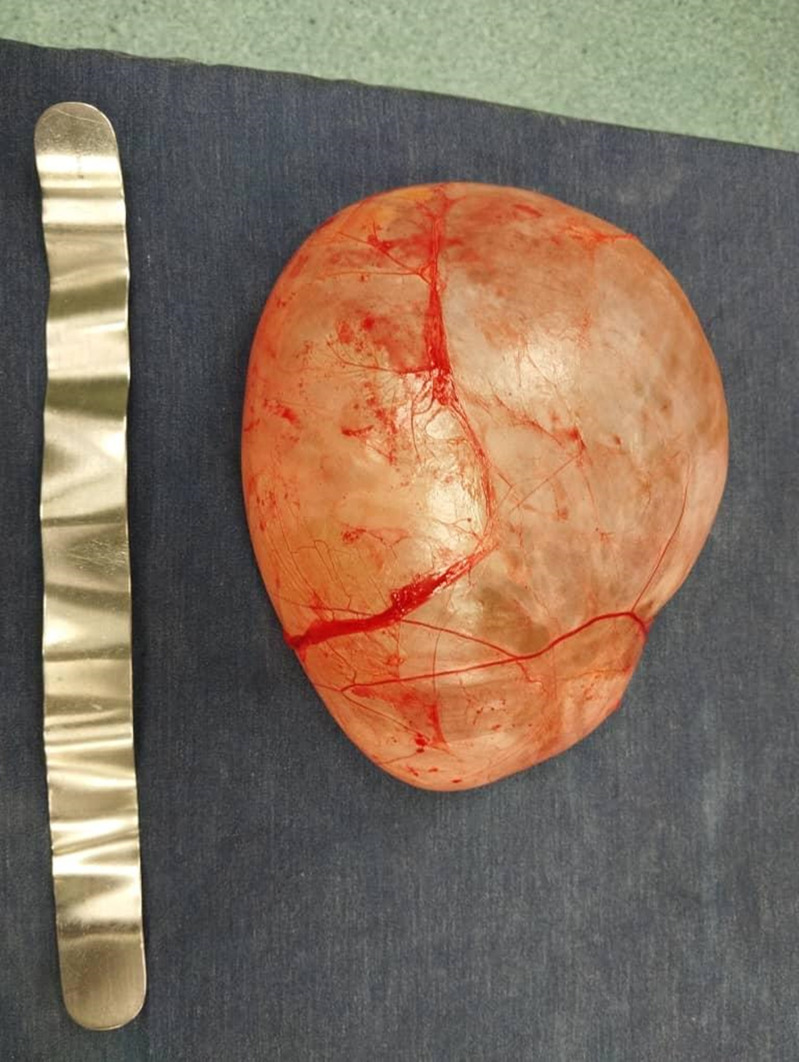
lymphangiome kystique abdominal après résection

**Figure 4:**
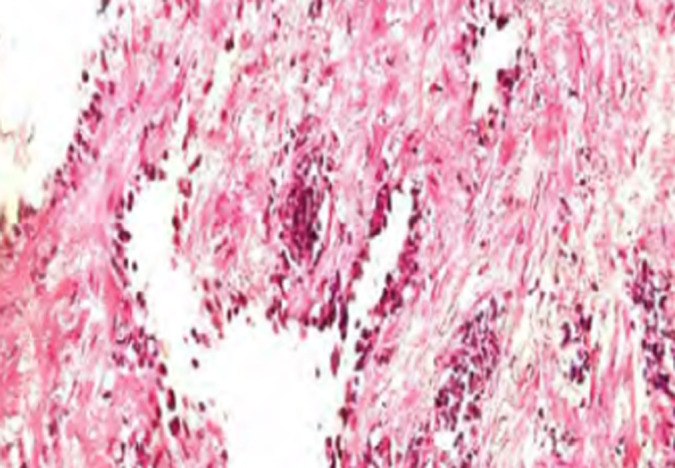
coupe histologique de la paroi kystique

**Intervention thérapeutique:** une laparotomie médiane est décidée. L'exploration peropératoire montre la présence d'une grosse masse liquidienne multikystique intrapéritonéale, extériorisée en partie à travers l'incision médiane et développée à partir du mésocolon gauche (Figure 2). Nous avons procédé à une dissection-résection du kyste dans sa totalité (Figure 3). L'examen anatomo-pathologique a permis de confirmer le diagnostic de lymphangiome kystique (Figure 4).

**Suivi et résultats des interventions thérapeutiques:** les suites post-opératoires sont simples, la patiente est sortie au 4^e^ jour post-opératoire. A un mois de suivi, la patiente est asymptomatique. Le contrôle radio-clinique à 3 et 6 mois est sans particularité.

**Perspectives du patient:** pendant le traitement, la patiente est satisfaite du niveau de soins qui lui a été prodigué.

**Consentement éclairé:** un consentement éclairé écrit est obtenu de la patiente pour participer à notre étude.

## Discussion

Les LK du mésocolon sont des localisations rares de l'adulte dont la conduite thérapeutique est complexe pouvant nécessiter des résections coliques [1]. Elles touchent aussi bien les hommes que les femmes avec une prédominance féminine [2]. La physiopathologénie du LK résulte d'une anomalie congénitale de connexion des canaux lymphatique et le réseau veineux responsable de lymphangiectasies, puis de formation de masses kystiques [3,4]. La symptomatologie clinique des LK est très diversifiée. Le maitre symptôme est la douleur abdominale et/ou une masse abdominale [2,4] comme ce fût le cas pour notre patiente. Enfin, il existe une variété clinique très rare, sous forme d'une carcinose péritonéale, appelée lymphangiomatose kystique péritonéale [3]. Le diagnostic du LK est basé essentiellement sur l'imagerie. En pratique, l'échographie est l'examen demandé en première intention, elle montre une masse kystique uni ou multiloculaires, à parois fines, hypoéchogène et des septas plus ou moins vascularisés en doppler couleur [5-7]. La TDM avec injection de produit de contraste est l'examen de référence [8,9]. Elle met en évidence une masse kystique homogène, hypodense, à cloisons fines ne prenant pas le produit de contraste. Elle permet aussi d'apprécier les rapports de la tumeur avec les organes de voisinage [1,6]. L'imagerie par résonance magnétique (IRM) est pratiquée en seconde intention. Elle permet en période préopératoire avec séquence vasculaire de mieux préciser les rapports de la tumeur avec les vaisseaux mésentériques. En revanche, l'exploration peropératoire permet de prédire le caractère résécable ou non de la tumeur. Néanmoins, la certitude diagnostic est apportée par l'étude anatomopathologique [2,6,7,9]. Macroscopiquement, le lymphangiome kystique peut être unique ou polykystique. Microscopiquement trois critères sont indispensables au diagnostic: 1) l'existence d'une tumeur kystique; 2) les cloisons sont constituées d'un stroma conjonctif riche en tissu lymphoïde; 3) le kyste est bordé d'un revêtement endothélial de type lymphatique [1,3,6,9]. Le traitement de référence des LK est la résection chirurgicale consistant en une exérèse complète de la tumeur kystique afin d'éviter toute récidive, par laparotomie ou laparoscopie [5-7,9].

Elle est indiquée lorsque la masse est symptomatique permettant d'éviter les complications et lorsqu'une complication est évoquée (hémorragie, torsion, infection). Néanmoins, la chirurgie à ciel ouvert est largement pratiquée par la majorité des auteurs comme dans notre cas. Lorsque la tumeur kystique adhère intimement à la racine du mésocolon ou du mésentère, la résection du côlon sous-jacent est nécessaire [2,5,6]. Dans notre cas, la dissection entre la paroi du kyste et celle du mésocolon s'est déroulée sans incidents peropératoires. L'évolution de la patiente est tout à fait favorable à un mois de l'intervention. Dans la littérature le risque de récidive des LK est de l'ordre de 10 à 15% [8-10]. Après 12 mois de suivi postopératoire, nous signalons aucun signe de récidive chez notre patiente. En revanche, le recul est assez faible pour pouvoir porter un pronostic à long terme.

## Conclusion

Le lymphangiome kystique du mésocolon est une pathologie bénigne malformative rare. Le diagnostic est évoqué par l'imagerie et confirmé par l'histologie. Une exérèse chirurgicale complète du kyste permet d'éviter la survenue de récidive. Le pronostic est généralement favorable avec une morbidité faible.
